# OLIF versus MI-TLIF for patients with degenerative lumbar disease: Is one procedure superior to the other? A systematic review and meta-analysis

**DOI:** 10.3389/fsurg.2022.1014314

**Published:** 2022-10-13

**Authors:** Jianqiang Wang, Jingwei Liu, Yong Hai, Yiqi Zhang, Lijin Zhou

**Affiliations:** ^1^ Department of Orthopedic Surgery, Beijing Chao-Yang Hospital, Capital Medical University, Beijing, China; ^2^ Department of Orthopedic Surgery, Beijing Hospital, National Center of Gerontology, Institute of Geriatric Medicine, Chinese Academy of Medical Sciences, Beijing, China

**Keywords:** degenerative lumbar disease, OLIF, MI-TLIF, meta-analysis, systematic review

## Abstract

**Purpose:**

To compare the effectiveness and safety of oblique lateral interbody fusion (OLIF) and minimally invasive transforaminal lumbar interbody fusion (MI-TLIF) for degenerative lumbar disease.

**Methods:**

We searched relevant studies in Embase, PubMed, Cochrane, and Web of Science databases comprehensively from inception to March 2022. The data were extracted from included studies, including operation indications, radiographic parameters, and clinical outcomes. Random or fixed-effects models were used in all meta-analyses according to the between-study heterogeneity.

**Results:**

In total, 30 studies, including 2,125 patients, were included in this meta-analysis. Our study found similar disk height, length of hospital stay, visual analog scale (VAS), and Oswestry disability index(ODI) between the two groups. However, the OLIF showed an advantage in restoring lumbar lordotic angle compared with MI-TLIF, with the pooled mean change of 17.73° and 2.61°, respectively. Additionally, the operative time and blood loss in the OLIF group appeared to be less compared with the MI-TLIF group. Regarding complications, the rates of the two groups were similar (OLIF 14.0% vs. MI-TLIF 10.0%), but the major complications that occurred in these two procedures differed significantly.

**Conclusion:**

The results of disk height, length of hospital stay, VAS, and ODI between the OLIF and MI-TLIF groups were similar. And the OLIF was superior in restoring lumbar lordotic angle, operative time, and blood loss. However, the OLIF group's complication rate was higher, although not significantly, than that in the MI-TLIF group.

## Introduction

With the increased human life expectancy around the world, the degenerative lumbar disease has a higher incidence, and it has become the leading cause affecting the quality of life in the elderly population (1). For patients with chronic low back pain or when the conservative treatment is ineffective, lumbar interbody fusion (LIF) is considered the most effective surgical procedure for degenerative lumbar disease (2), including degenerative spondylolisthesis, disc herniation, and deformity. Spinal fusion surgery can be performed to restore the disc height (DH) and immobilize unstable segments (3). According to different surgical approaches, LIF can be traditionally divided into the anterior, posterior, and open transforaminal types (4).

Although the classical surgical method can achieve satisfactory clinical efficacy, such iatrogenic complications as excessive blood loss, nerve injury, and muscular denervation induced by paravertebral muscle stripping cannot be avoided (5, 6). Besides, this surgical method also has the disadvantages of longer operative time and hospital stays. In recent years, minimally invasive techniques have achieved significant advancement, which contributes to fewer surgical complications and shorter hospital stays (6). Foley and Lefkowitz (7) proposed minimally invasive transforaminal lumbar interbody fusion (MI-TLIF) in 2002, which could minimize operative trauma compared with traditional open transforaminal LIF. In addition, Silvestre described oblique lateral LIF in 2012 to avoid nerve injury (8).

MI-TLIF and oblique lateral interbody fusion (OLIF) have achieved favorable efficacy in extensive clinical applications. However, the differences between OLIF and MI-TLIF have not been sufficiently explored. OLIF is a minimally invasive LIF with a surgical approach to the natural space in the lateral front of the body without damaging the muscle, ligament, and bone structure (8). MI-TLIF has a surgical approach through the intervertebral multifidus and longissimus muscle system of the lumbar paraspinal, which does not require extensive dissection. The intermuscular approach is characterized by less injury and less bleeding, and excessive traction of nerve roots and dual sacs would not be required for this approach, so it is safer to handle the intervertebral space (7). However, it remains unclear about the optimal surgical technique for treating these diseases. Therefore, to compare the radiographic and clinical outcomes of OLIF and MI-TLIF in treating degenerative lumbar disease, some relevant studies in recent years were summarized in the meta-analysis, thus providing the latest and most compelling evidence for clinicians.

## Methods

### Retrieval strategy and literature selection

The systematic review and meta-analysis comparing OLIF and MIS-TLIF in treating degenerative lumbar disease were conducted in accordance with the Preferred Reporting Items for Systematic Reviews and Meta-Analyses (PRISMA) Statement.

In the study, Embase, PubMed, Cochrane, and Web of Science were comprehensively retrieved to select relevant articles published from January 2019 to March 2022 based on an English language restriction condition. The combined text and MeSH terms included (“OLIF” or “oblique lumbar interbody fusion”), (“MIS-TLIF” or “minimally invasive transforaminal lumbar interbody fusion”), and (“degenerative scoliosis” or “degenerative spinal deformity”). Moreover, the reference lists were also checked by manual retrieval for relevant articles.

### Inclusion and exclusion criteria

Two investigators independently conducted screening in respect of the title and abstract of articles, and the full text of relevant articles was reappraised according to the inclusion and exclusion criteria. A third investigator participated in the resolution in case of any disagreement. The inclusion criteria included: (1) target patients: a diagnosis of degenerative lumbar disease; (2) intervention: single spine surgery, OLIF or MI-TLIF; (3) outcomes: sufficient information including surgical indications (operative blood loss, operative time, and length of hospital stay), radiographic parameters (DH and lumbar lordotic angle [LLA]), visual analog scale (VAS), and Oswestry disability index (ODI). Reviews and case reports were not included in the meta-analysis. Duplicates or multiple publications of the same study were also excluded.

### Data extraction and literature quality evaluation

According to an established data abstraction form, two investigators independently extracted the following data from each included article: (1) study characteristics (author, year of publication, patient diagnosis, and number of patients); (2) radiographic parameters (DH and LLA); (3) surgical indications (operative blood loss, operative time, and length of hospital stay); (4) VAS and ODI.

Two investigators independently evaluated the literature quality according to the PRISMA recommendation. These studies were evaluated based on the Newcastle-Ottawa scale (NOS) (9), which was composed of nine items, including selection (four points), comparability (two points), and exposure (three points). A study awarded seven or more points can be considered high quality. The author’s name, institution, journal name, and other related information were hidden during the evaluation process to reduce the influence of subjective factors.

### Data analysis

All statistical analyses were performed with Stata software (version 14.0). The heterogeneity among these studies was evaluated with the Cochran Q and *I*^2^ test (10). A fixed effects model was used if *P* > 0.05 or *I*^2^ < 50%; otherwise, a random effects model was employed (11). Additionally, a meta-regression model was used to investigate the contribution of age and follow-up duration to the radiographic and clinical outcomes. *P*-value <0.05 was considered statistically significant.

## Results

### Literature retrieval

**Figure 1:**
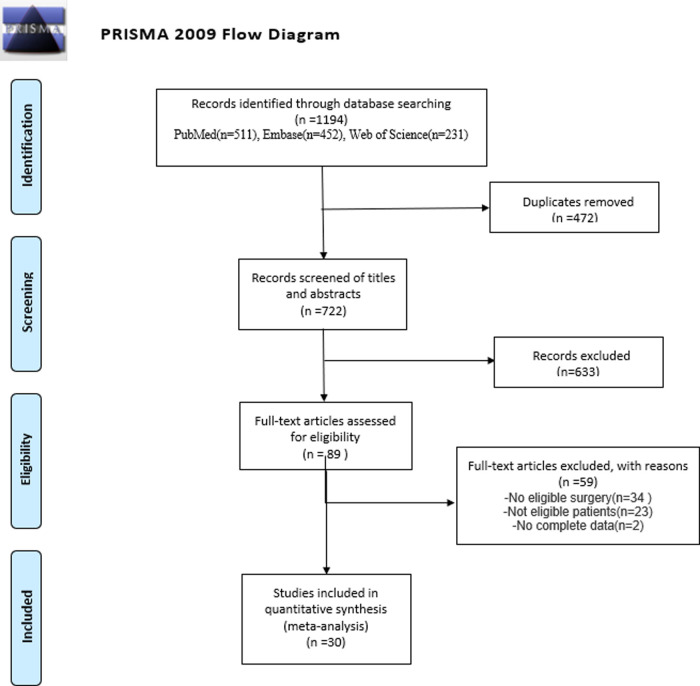
Flow diagram illustrating number of studies evaluated at each stage in the meta-analysis.

In this study, a total of 1,194 studies were screened from Embase, PubMed, Cochrane, and Web of Science. After duplicates were removed, 633 studies were involved in case reports, irrelevant studies, and reviews, and hence were excluded. Two investigators conducted a full-text assessment for the remaining 89 articles. Eventually, only 30 studies including 2,125 patients in total were eligible and included in this meta-analysis (Figure 1**)**.

### Characteristics of included studies

In this meta-analysis, a total of 30 studies were included. Among them, there were 15 retrospective pre-post studies related to the clinical efficacy of OLIF, 12 studies related to the clinical efficacy of MI-TLIF, and 3 retrospective cohort studies related to the comparison of the efficacy between OLIF and MI-TLIF. Specifically, 1,057 patients aged 50.8–69.7 years (mean 59.2 y) in 18 studies received OLIF, and 1,068 patients aged 50.9–66.4 years (mean 59.3 y) in 15 studies received MI-TLIF. The basic characteristics of these studies are listed in Tables 1, 2.

**Table 1:** Characteristics of OLIF studies.

Study	Year	Country	Sample size	Age,years (Mean ± SD)	Gender (Male/Famle)	Follow-up, months (range)	Hospital stay (days) Mean (SD)	Operative time (min) Mean (SD)	Blood loss (ml) Mean (SD)
Abe (12)	2016	Japan	155	63.5 ± 17	69/86	≥1	—	—	—
Chen (13)	2018	China	34	66 ± 11	12/22	≥12	—	163 (68)	116 (148)
Cho (14)	2020	Korea	28	69.7 ± 6.9	9/19	≥12	—	165.1 (44.4)	190.6 (69.6)
Heo (15)	2017	Korea	14	66.3 ± 8.8	6/8	≥12	—	155.8 (45.1)	105.5 (20.9)
Jin C (16)	2018	Korea	29	60.1 ± 4.2	10/19	≥12	6.8 (6.6)	122 (97)	253.4 (120.7)
Jun (17)	2017	Japan	20	69 ± 7.8	9/11	≥6	—	—	—
Kim (18)	2017	Korea	32	68 ± 5.0	4/28	≥24	—	98.3 (8.5)	99.1 (5.0)
Li (19)	2021	China	28	57.5 ± 10.4	7/21	≥6	2.8 (1.2)	186.44 (36.5)	55.9 (57.4)
Liu (20)	2020	China	108	50.8 ± 6.25	46/62	≥12	4.8 (1.9)	92 (34)	48 (15)
Mun (21)	2020	Korea	74	64.1 ± 9.3	20/54	≥12	—	167.7 (24.9)	92 (41.8)
Ohtori (5)	2015	Japan	35	67 ± 6.5	17/18	≥7	—	—	—
Poppenborg (22)	2020	Germany	157	62 ± 15	45/73	≥12	—	170.3 (59.7)	—
Sheng (23)	2020	China	38	65.29 ± 8.88	8/30	≥12	5.2 (1.3)	90.8 (7.9)	63.9 (23.3)
Shunsuke (24)	2015	Japan	28	65.3 ± 17.6	10/18	≥3	—	72.5 (21.0)	—
Xi (25)	2020	China	25	55.12 ± 16.88	7/18	≥12	5 (2.9)	154.9 (64.7)	74 (43.6)
Yang (26)	2020	China	11	62.37 ± 11.78	7/4	≥3	—	127.3 (21.5)	115.5 (19.2)
Zairi (27)	2017	Canada	6	61.8 ± 6.0	3/3	≥12	—	275.8 (65.8)	283.3 (112.5)
Zeng (28)	2018	China	235	61.9 ± 0.21	79/156	≥12	—	115 (66)	120 (72.5)

**Table 2:** Characteristics of TLIF studies.

Study	Year	Country	Sample size	Age, years (Mean ± SD)	Gender (Male/Famle)	Follow-up, months (range)	Hospital stay (days) Mean (SD)	Operative time (min) Mean (SD)	Blood loss (ml) Mean (SD)
Chen (13)	2018	China	39	66 ± 12	19/20	≥12	—	233 (79)	434 (201)
Fan (29)	2016	China	24	65.9	14/10	≥12	12.5 (2.8)	270.8 (33.7)	666.7 (314.3)
Gu (30)	2014	China	44	66.4 ± 6.7	19/25	≥12	9.3 (3.7)	195.5 (28)	248.4 (943)94.3
Hamid (31)	2017	Singapore	56	53.7 ± 11.3	30/40	24	2.8 (11)	167 (49)	126 (107)
Lee K (32)	2012	Singapore	72	52.2 ± 13.8	20/52	≥2	3.2 (2.9)	166.4 (52.1)	50.6 (161)
Lee W (33)	2016	Korea	70	63.41 ± 10.3	24/46	≥12	10.8 (5.39)	197.6 (45.9)	735.3 (462.1)
Li (19)	2021	China	35	59.3 ± 9.86	8/27	≥6	3.7 (0.79)	199 (59.6)	190 (66.3)
Min (34)	2013	Korea	172	56.78 ± 13	45/78	≥2	—	—	—
Park (35)	2014	Korea	124	59.3 ± 14.7	45/79	24	7.9 (6.1)	183.9 (37.3)	250.1 (192.5)
Parker (36)	2012	USA	15	50.8 ± 7.9	7/8	≥2	3 (0.5)	300 (50)	200 (31.3)
Sheng (23)	2020	China	55	60.62 ± 12.3	25/30	≥12	7.2 (1.6)	100.2 (14.59)	186.4 (80.2)
Wale (37)	2014	USA	57	61.1	17/40	24	3.6 (1.0)	161 (7.6)	95 (20)
Wang (38)	2014	China	204	52.4 ± 10.1	98/106	≥1	—	—	—
Wu (39)	2018	China	79	58.1 ± 12.8	33/46	24	5.8 (1.4)	145.5 (21.5)	163.7 (49.6)
Zhao (40)	2018	China	22	63.7 ± 8	8/14	≥2	5.4 (0.9)	153.3 (26.3)	175 (83.4)

### Literature quality evaluation and publication bias

These 30 studies were independently evaluated by two investigators from the perspective of the risk of bias according to the NOS, their scores were all ≥6 points (Table 3). All studies included appropriate patients with a clear diagnosis and presented important outcomes after OLIF or MI-TLIF.

**Table 3:** Quality assessment for the included studies according to Newcastle-Ottawa scale (NOS).

Study	Year	*S*				*C*		*E*			Total score
		*S1*	*S2*	*S3*	*S4*	*C1*	*C2*	*E1*	*E2*	*E3*	
OLIF											
Abe (12)	2016	★	★	★	–	★	–	★	–	★	6
Chen (13)	2018	★	★	★	–	★	–	★	★	★	7
Cho (14)	2020	★	★	★	–	★	★	★	★	★	8
Heo (15)	2018	★	★	★	–	★	–	★	★	★	7
Jin (16)	2018	★	★	★	–	★	★	★	★	★	8
Jun (41)	2017	★	★	★	–	★	★	★	★	★	8
Kim (18)	2017	★	★	★	–	★	–	★	★	★	7
Li (19)	2021	★	★	★	–	★	★	★	★	★	8
Liu (20)	2020	★	★	★	–	★	★	★	★	★	8
Mun (21)	2020	★	★	★	–	★	–	★	★	★	7
Ohtori (5)	2015	★	★	★	–	★	–	★	–	★	6
Poppenborg (22)	2020	★	★	★	–	★	★	★	★	★	8
Sheng (23)	2020	★	★	★	–	★	★	★	★	★	8
Shunsuke (24)	2015	★	★	★	–	★	★	★	–	★	7
Xi (25)	2020	★	★	★	–	★	–	★	★	★	7
Yang (26)	2020	★	★	★	–	★	★	★	–	★	7
Zairi (27)	2017	★	★	★	–	★	–	★	–	★	6
Zeng (28)	2018	★	★	★	–	★	★	★	★	★	8
TLIF											
Fan (29)	2016	★	★	★	–	★	★	★	★	★	8
Gu (30)	2014	★	★	★	–	★	–	★	★	★	7
Hamid (31)	2017	★	★	★	–	★	★	★	★	★	8
Lee K (32)	2012	★	★	★	–	★	–	★	★	★	7
Lee W (33)	2016	★	★	★	–	★	–	★	★	★	7
Min (34)	2013	★	★	★	–	★	–	★	★	★	7
Park (35)	2014	★	★	★	–	★	–	★	★	★	7
Parker (36)	2012	★	★	★	–	★	★	★	★	★	8
Wale (37)	2014	★	★	★	–	★	★	★	★	★	8
Wang (38)	2014	★	★	★	–	★	★	★	–	★	7
Wu (39)	2018	★	★	★	–	★	★	★	★	★	8
Zhao (40)	2018	★	★	★	–	★	★	★	★	★	8

***S*** Selection, ***C*** Comparability, ***E*** Exposure, ***S*1** Representativeness of the exposed cohort, ***S*2** Selection of the non-exposed cohort, ***S*3** Ascertainment of exposure, ***S*4** Demonstration that outcome of interest was not present at the start of the study. ***C*1** Comparability of controls for the most important factor, ***C*2** Comparability of controls for a second important factor. ***E*1** Assessment of the outcome, ***E*2** Was follow-up long enough for outcomes to occur, ***E*3** Adequacy of follow up of cohorts.

### Length of hospital stay

**Figure 2:**
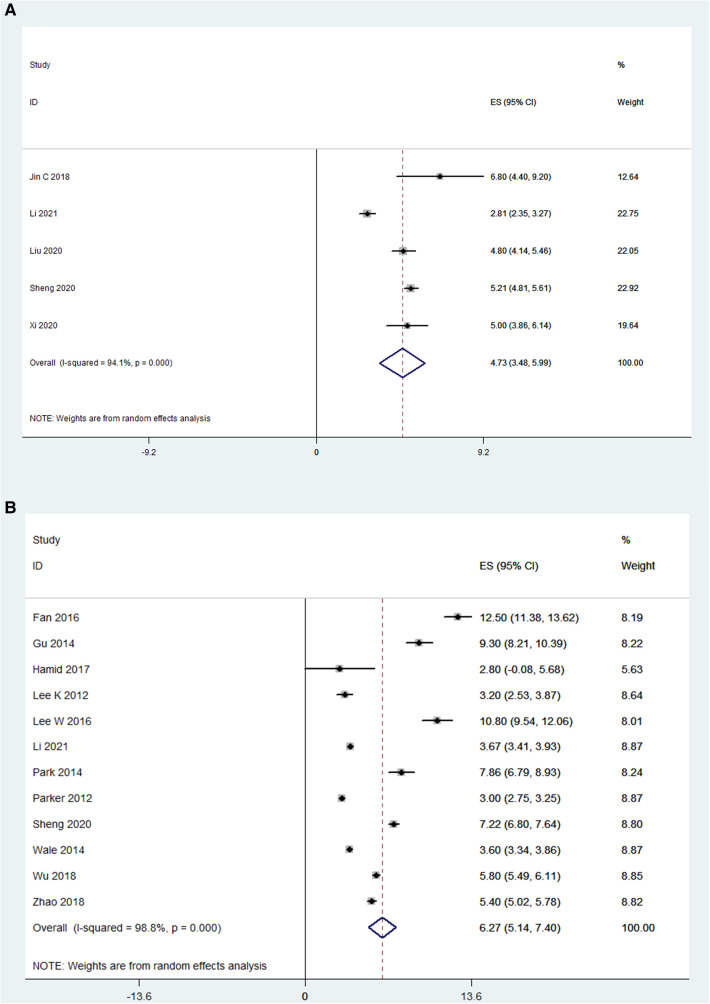
Forest plot and pooled estimates of the length of hospital stay. Outcomes assessed are (**A**) OLIF group; (**B**) MI-TLIF group.

For OLIF, the length of hospital stay was reported in 5 studies (*N* = 228), and the pooled mean length of hospital stay was 4.73 days [95% CI (3.48, 5.99), *P* < 0.001; Figure 2A]. There was substantial heterogeneity between studies (*I*^2^ = 94.1%). For MI-TLIF, the length of hospital stay was reported in 12 studies (*N* = 653), and the pooled mean length of hospital stay was 6.27 days [95% CI (3.48, 5.99), *P* < 0.001; Figure 2B].

### Operative time

**Figure 3:**
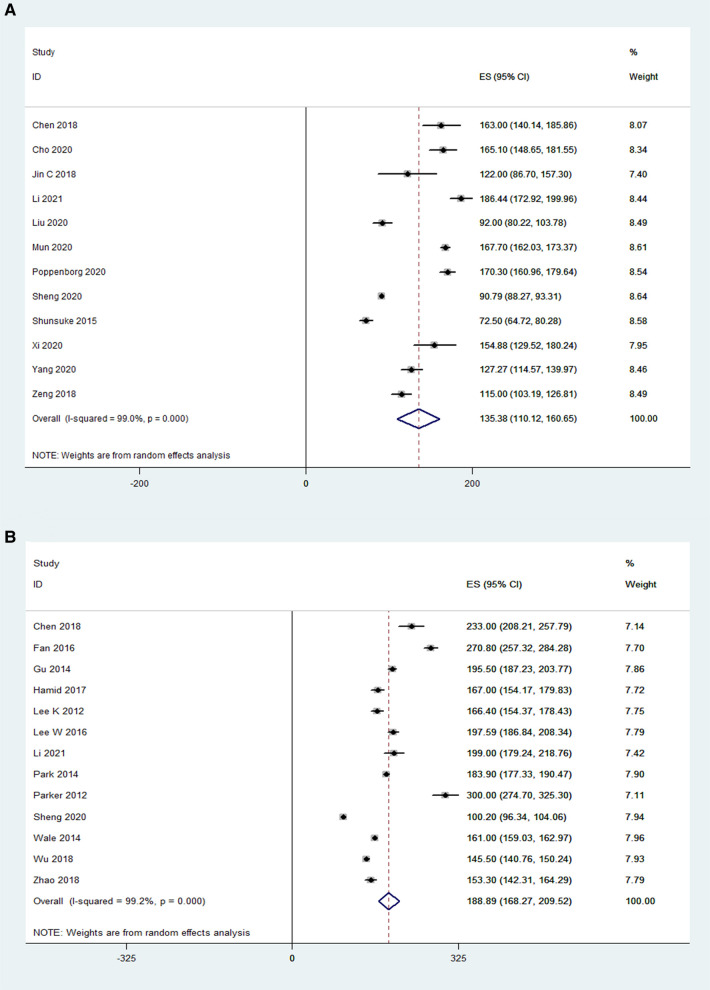
Forest plot and pooled estimates of operative time. Outcomes assessed are (**A**) OLIF group; (**B**) MI-TLIF group.

The operative time was reported in 12 OLIF studies (*N* = 795) and 13 MI-TLIF studies (*N* = 692). Based on the random effects model, the mean operative time was 135.4 min in the OLIF group [95% CI (110.12, 160.65), *P* < 0.001; Figure 3A], compared with 188.9 min in the MI-TLIF group [95% CI (168.27, 209.52), *P* < 0.001; Figure 3B].

### Blood loss

**Figure 4:**
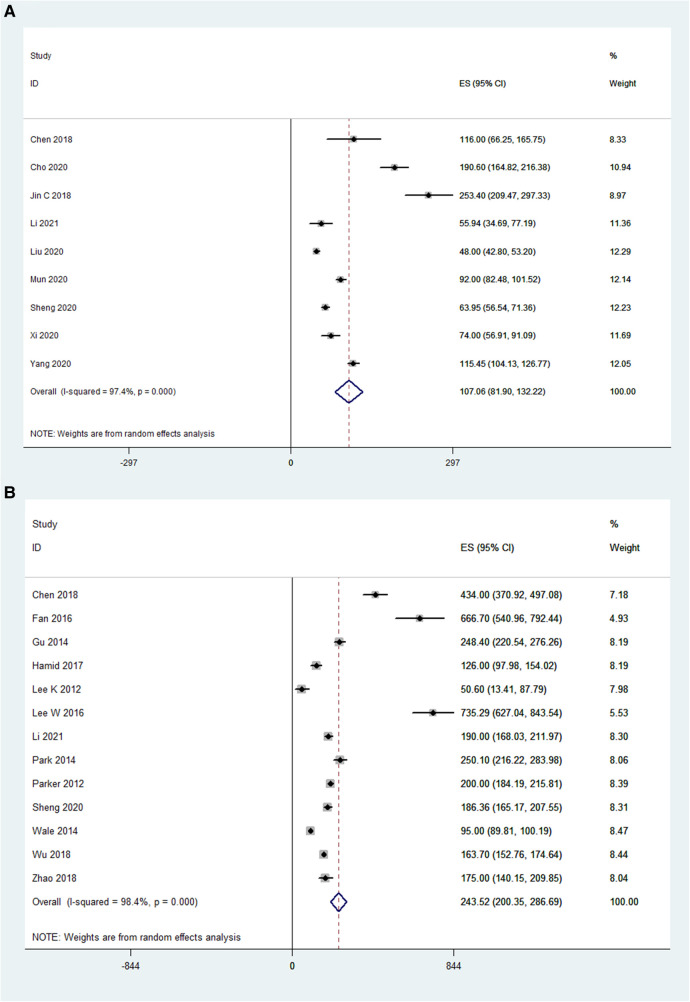
Forest plot and pooled estimates of blood loss. Outcomes assessed are (**A**) OLIF group; (**B**) MI-TLIF group.

Blood loss was reported in 10 OLIF studies (*N* = 610) and 13 MI-TLIF studies (*N* = 692). According to the pooled analysis results, the mean blood loss in the OLIF group was 107.1 ml [95% CI (81.90, 132.22), *P* < 0.001; Figure 4A], compared with 243.52 ml in the MI-TLIF group [95% CI (200.35, 286.69), *P* < 0.001; Figure 4B].

### Disc height

**Figure 5:**
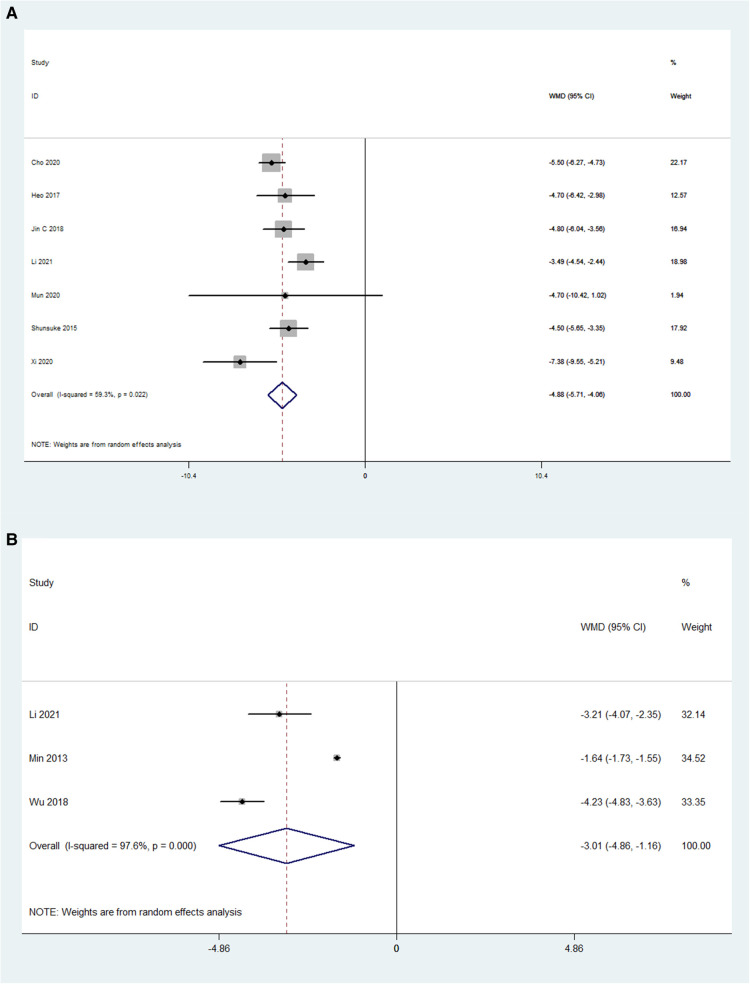
Forest plot and pooled estimates of disk height. Outcomes assessed are (**A**) OLIF group; (**B**) MI-TLIF group.

The pooled analysis results of 7 OLIF studies (N = 226) showed that OLIF induced a mean increase in the disc height, and the weighted mean difference (WMD) was −4.88 [95% CI (−5.71, −4.06), *P* < 0.001; Figure 5A]. While, the pooled analysis results of 3 MI-TLIF studies (*N* = 286) also showed a mean increase in the DH caused by MI-TLIF, and the weighted mean difference (WMD) was −3.01 [95% CI (−4.86, −1.16), *P* = 0.001; Figure 5B]. The surgical effect can be evaluated by subtracting the postoperative DH from the preoperative DH. Hence, larger negative values represented more significant surgical effects. Of note, before the pooled analysis of the DH, the number of operative levels between both study groups was compared. The results showed that the mean number of OLIF-operated levels was 1.31 ± 0.3, and that of MI-TLIF-operated levels was 1.29 ± 0.44. There was no significant difference between these surgery techniques (*P* = 0.55).

### Lumbar lordotic angle

**Figure 6:**
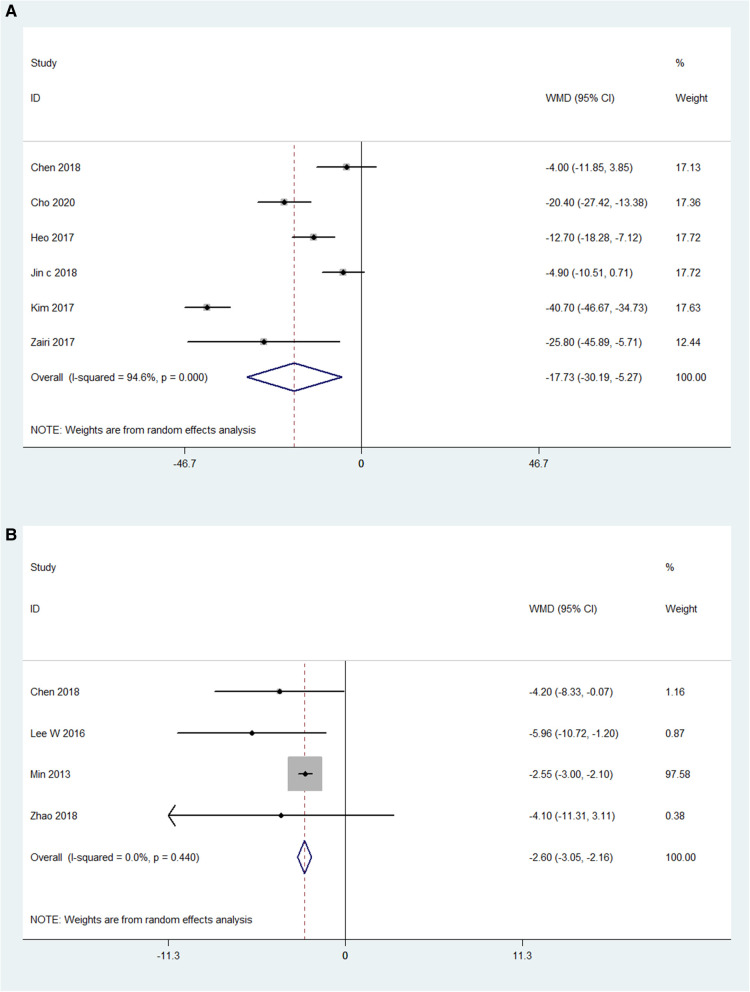
Forest plot and pooled estimates of lumbar lordotic angle. Outcomes assessed are (**A**) OLIF group; (**B**) MI-TLIF group.

The preoperative and postoperative LLAs were also compared in 6 OLIF studies (*N* = 133) and 4 MI-TLIF studies (*N* = 303). The pooled analysis results of these OLIF studies showed the WMD was −17.73 [95% CI (-30.19, −5.27), *P* < 0.001; Figure 6A]. The pooled analysis results of these MI-TLIF studies showed the WMD was −2.61 [95% CI (−3.05, −2.16), *P* < 0.001; Figure 6B]. The changes in the LLA can be evaluated by subtracting the postoperative LLA from the preoperative LLA. Hence, larger negative values represented more significant surgical effects. The WMD in both groups was positive, indicating that LLA was improved after surgery.

### Visual analog scale (VAS)

**Figure 7:**
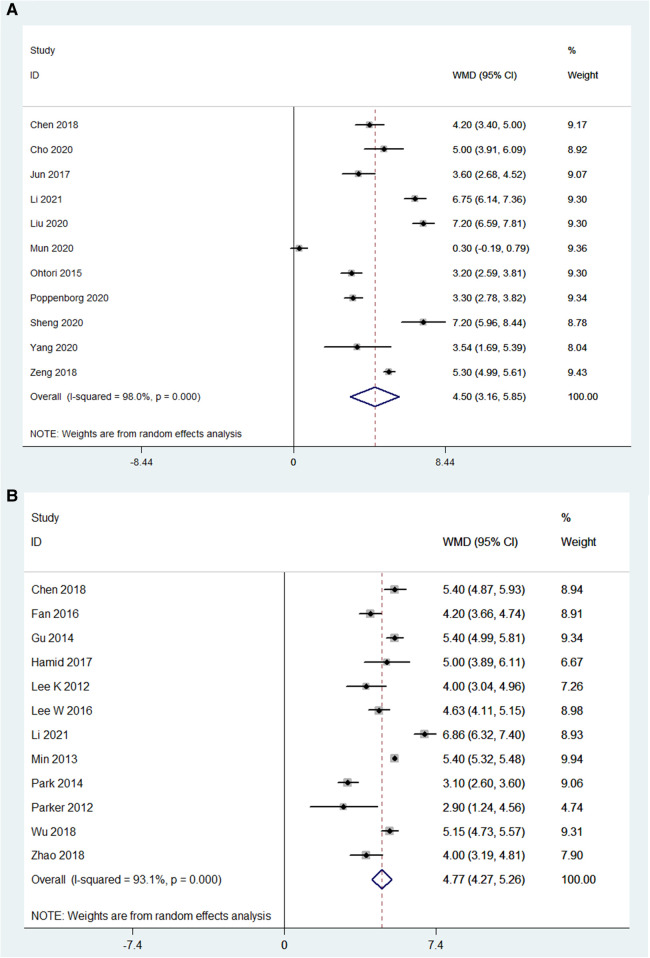
Forest plot and pooled estimates of visual analog scale. Outcomes assessed are (**A**) OLIF group; (**B**) MI-TLIF group.

For OLIF surgery, the VAS was reported in 11 studies (*N* = 692), and the preoperative VAS and postoperative VAS were compared in these studies (*I*^2^ = 98%). The pooled analysis results showed that the WMD was 4.51 [95% CI (3.16, 5.85), *P* < 0.001; Figure 7A]. For MI-TLIF surgery, the VAS was reported in 12 studies (*N* = 752). The pooled analysis results showed that there was a significant difference in VAS before and after MI-TLIF surgery [WMD = 3.24, 95% CI (0.22, 6.27), *P* < 0.001; Figure 7B]. In addition, the WMD in both groups was positive, indicating that the VAS score was improved after surgery.

### Oswestry disability Index (ODI)

**Figure 8:**
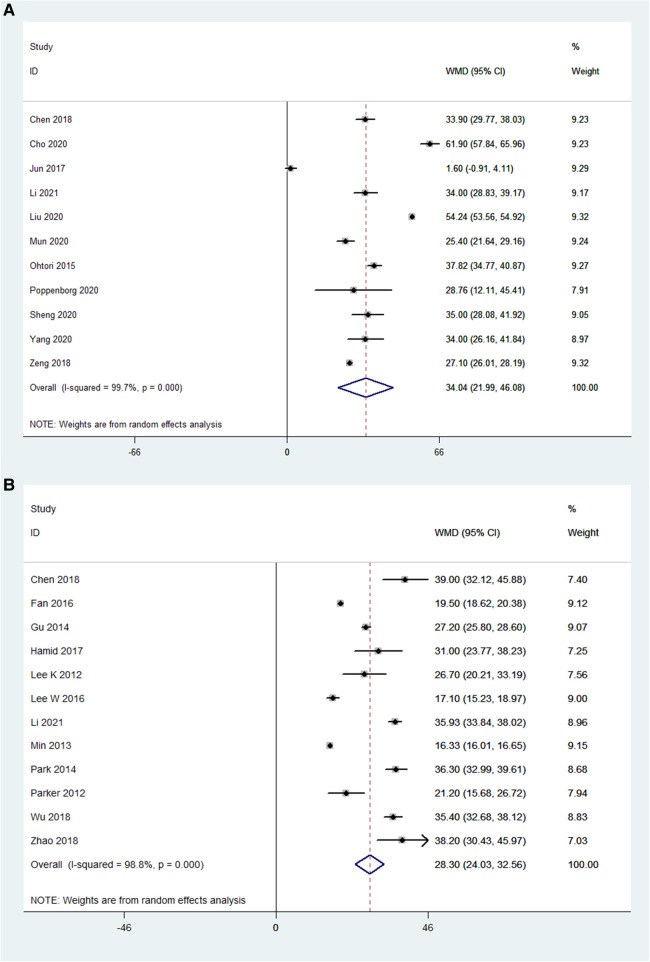
Forest plot and pooled estimates of Oswestry disability index. Outcomes assessed are (**A**) OLIF group; (**B**) MI-TLIF group.

The preoperative ODI and postoperative ODI were compared in 11 OLIF studies (*N* = 1,289). The pooled WMD was 34.05 [95% CI (21.99, 46.08), *P* < 0.001; Figure 8A]. While, the preoperative ODI and postoperative ODI were compared in 12 MI-TLIF studies (*N* = 752). The pooled WMD was 28.30 [95% CI (24.03, 32.56), *P* < 0.001; Figure 8B]. The improvement of ODI can be evaluated by subtracting the postoperative ODI from the preoperative ODI. Hence, larger positive values represented more significant surgical effects. The WMD in both groups was positive, indicating that ODI was improved after surgery.

### Complications

**Figure 9:**
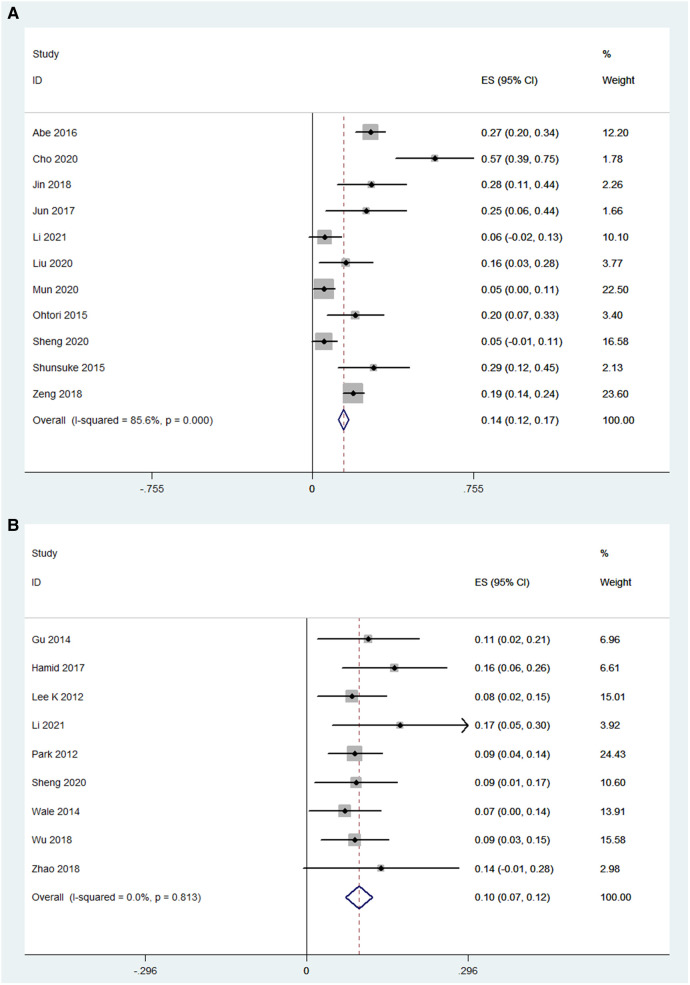
Forest plot and pooled estimates of complications. Outcomes assessed are (**A**) OLIF group; (**B**) MI-TLIF group.

The number of complications was reported in 11 OLIF studies (*N* = 726) (*I*^2^ = 85.6%). The pooled analysis results of these OLIF studies showed that the incidence of complications was 14.5% [95% CI (12.0%, 16.9%), *P* < 0.001], with individual study estimates ranging from 5.4% to 28.6% (Figure 9A). The most common complication was thigh pain/numbness (7.3%). Other main complications included endplate injury (5.4%), vascular injury (2.5%), and neurological injury (1.0%). The details of perioperative complications of OLIF are listed in Table 4. While, the number of complications was reported in 9 MI-TLIF studies (*N* = 544), accounting for a pooled prevalence of 10.0% [95% CI (7.1%, 12.3%), *P* < 0.001], with individual study estimates ranging from 7.2% to 17.1% (Figure 9B). The most common complication was endplate injury (3.41%). Other main complications included neurological injury (1.0%), wound infection (0.85%), and thigh pain/numbness (0.61%). The details of perioperative complications of MI-TLIF are also listed in Table 4.

**Table 4:** Complications of OLIF and MI-TLIF studies.

Study (OLIF)	Number of complications	Major complication	Number of patients
Abe 2016	42	Transient thigh pain/numbness	155
Cho 2020	16	Endplate injury	28
Jin 2018	8	Transient thigh pain/numbness	29
Jun 2017	5	Transient thigh pain/numbness	20
Li 2021	2	Transient thigh pain/numbness	35
Liu 2020	5	Endplate Injury/Psoas weakness	32
Mun 2020	4	Vascular injury	74
Ohtori 2015	7	Transient thigh pain/numbness	35
Sheng 2020	3	Psoas weakness	55
Shunsuke 2015	8	Transient thigh pain/numbness	28
Zeng 2018	45	Endplate injury	235
**Study (MI-TLIF)**	**Number of complications**	**Major complication**	**Number of patients**
Gu 2014	5	Wound infection	44
Hamid 2017	9	Endplate injury	56
Lee K 2012	6	Endplate injury	72
Li 2021	6	Wound infection	35
Park 2014	11	Endplate injury	124
Sheng 2020	5	Transient thigh pain/numbness	55
Wale 2014	4	Endplate injury	57
Wu 2018	7	Transient thigh pain/numbness	79
Zhao 2018	3	Transient thigh pain/numbness	22

### Meta-regression analyses

The meta-regression analysis results showed that the age of patients would not affect the DH, LLA, VAS, and ODI scores in both OLIF and MI-TLIF groups. However, the follow-up duration was associated with the DH and postoperative ODI scores in the OLIF group (Table 5).

**Table 5:** Meta-Regression analysis on follow-Up and mean age.

	OLIF group		MI-TLIF group	
	Age (*P* > t)	Follow-up duration (*P* > t)	Age (*P* > t)	Follow-up duration (*P* > t)
Disk height	0.02 (0.99)	−2.68 (0.044)*	−2.09 (0.28)	−0.82 (0.564)
Lumbar lordotic angle	0.16 (0.88)	−1.10 (0.35)	−0.17 (0.89)	−0.14 (0.76)
Visual analog scale	−1.69 (0.13)	0.67 (0.52)	0.86 (0.41)	−0.76 (0.47)
ODI scores	−1.52 (0.17)	2.48 (0.038)*	0.29 (0.78)	0.36 (0.73)

* *P* < 0.05 was considered the factor contributing to the heterogeneity of effect.

## Discussion

The optimal surgical procedure for treating degenerative lumbar disease has not been established for years. There are various disadvantages for traditional open surgical methods (for instance anterior, posterior, and transforaminal LIF), such as more iatrogenic complications, longer operative time, and longer hospital stays. Thus, OLIF and MI-TLIF can be widely used in treating degenerative lumbar disease with the significant advancement of minimally invasive surgical techniques. As per many studies (28, 41), both OLIF and MI-TLIF can achieve satisfactory clinical outcomes. However, the effectiveness and safety of both surgical techniques remain controversial and have not been systematically confirmed. Thus, relevant studies in the last three years were included in this study to evaluate and compare the effectiveness and safety of OLIF and MI-TLIF during the treatment of degenerative lumbar disease, thus providing the latest and most compelling evidence for clinicians. In the past three years, MIS TLIF and OLIF have achieved rapid and significant development with the advancement of instruments, the increased quantity of surgery, the popularization of techniques, and the popularization of training courses. Compared with conventional surgical techniques, there was no update on the general review. In the complete literature review, only the articles published in the last three years were included and updated for analysis.

### Surgical indications

Among these included studies, the length of hospital stay was evaluated in 5 OLIF studies and 12 MI-TLIF studies. There was a similar length of hospital stay between the OLIF (4.73 days) and MI-TLIF (6.27 days) groups. As is reported in some studies (42, 43), OLIF can reduce the length of hospital stay compared with conventional surgical techniques, such as posterior LIF. Nevertheless, there was no significant difference between OLIF and minimally invasive procedures, such as MI-TLIF (44, 45). This result was consistent with our findings. In one recent meta-analysis (46), the length of stay in the MI-TLIF group was significantly shorter than that in the Open-TLIF group. However, Sulaiman et al. (37) and Lau et al. (47) found no significant difference between the two groups. Besides, Hey et al. (48) also found no significant difference in the length of hospital stay between Open-TLIF and MI-TLIF at a single level.

The operative time and blood loss in the OLIF group appeared to be less than those in the MI-TLIF group. According to a study of Jin et al. (49), it may be attributed to a smaller surgical incision due to the muscle-splitting approach in the OLIF, which may result in shorter operative time. In addition, the retroperitoneal space was reached by blunt dissection during OLIF, which may lead to less bleeding. However, the screw placement and combination at multiple levels during MI-TLIF may need much operative time.

### Radiographic parameters

To evaluate the improvement in radiographic parameters by OLIF and MI-TLIF, the DH and LLA in these studies were analyzed. The analysis results showed that OLIF and MI-TLIF achieved similar improvement in the DH, with the pooled mean change being 4.88 and 3.01 mm, respectively. Besides, the postoperative DH in both groups was not significantly higher than the preoperative one. This finding was consistent with the results of previous studies, which indicated that OLIF and MI-TLIF might not restore the DH significantly (13, 50).

Moreover, the pooled analysis results showed that the OLIF group achieved more significant improvement in the LLA than the MI-TLIF group, with the pooled mean change being 17.73° and 2.61°, respectively. This result partly differed from that of some previous studies. In a recent review, Li et al. (51) reported high similarities in the restoration of LLA in both groups. The difference can be explained in several ways. According to the findings of Sato et al. (17), LLA can be restored effectively by OLIF with posterior supplement fixation. In this study, most patients had undergone posterior supplement fixation. Besides, the OLIF technique can more easily reach the anterior column and might use cages with higher lordotic angles (5).

### Clinical outcomes

In this study, VAS and ODI were employed to measure clinical outcomes. The pooled analysis results showed that both OLIF and MI-TLIF can significantly decrease VAS (for back pain) and ODI scores. There were similar VAS results between the OLIF and MI-TLIF groups (4.51 vs. 3.24). In theory, however, OLIF would significantly reduce the postoperative VAS score due to minor trauma compared with MI-TLIF. The use of analgesics may partially contribute to similar results. Furthermore, it was also found that the ODI scores in the OLIF group had a more significant decrease compared with the MI-TLIF group (34.05 vs. 28.30).

### Complications

According to the meta-analysis results, there was no significant difference in the incidence of complications between the OLIF group (14.0%) and the MI-TLIF group (10.0%). However, the major complications that occurred in these two procedures differed significantly. The most common complication of OLIF was thigh pain/numbness (7.3%), and other main complications included hardware failure (5.4%), vascular injury (2.5%), and neurological injury (1.0%). This result was consistent with that of a previous study (52). Considering the anatomy of the psoas muscle (complicated nerve plexuses), it can be prone to damage these nerve plexuses during the transpsoas approach. Of course, the incidence of thigh pain/numbness was lower than that in those traditional procedures, such as LLIF (53). Most of these complications are transient and can recover during the follow-up period. While, the most common complication of MI-TLIF was hardware failure (3.41%), and other main complications included neurological injury (1.0%), wound infection (0.85%), and thigh pain/numbness (0.61%). According to a study of Zeng et al. (28), endplate injury, cage subsidence, and shifting can be induced by multiple factors, such as over-distraction and aggressive endplate reaming. Besides, obesity and osteoporosis could also partially explain the endplate-related complications in these two surgery techniques (54). Furthermore, it had been reported that posterior fixation, which can enhance segmental stability, should be applied in case of endplate injury (55). Overall, these surgical procedures should be selected properly, and imaging guidance with high accuracy may be helpful.

### Limitations

There are several highlights in this meta-analysis, such as containing the latest studies in the last three years and analyzing the complications in detail. However, some limitations should also be mentioned. Firstly, most of these included studies are short of a controlled group, and hence it is difficult to directly compare the effect or complications between OLIF and MI-TLIF. Besides, posterior fixation was applied in some patients, which may affect the reliability of the conclusion.

## Conclusion

The OLIF and MI-TLIF can achieve similar results in the length of hospital stay, DH, and VAS. OLIF is superior to MI-TLIF in respect of operative time, blood loss, LLA restoration, and ODI scores. Moreover, although the incidence of complications is similar between OLIF and MI-TLIF, there are significant differences in the main complications between both surgical techniques.

## Data Availability

The raw data supporting the conclusions of this article will be made available by the authors, without undue reservation.
